# Teaching technological surgeries: The art of integrating technique, technology, skill, and didactic methods

**DOI:** 10.1016/j.clinsp.2023.100211

**Published:** 2023-05-17

**Authors:** Newton Kara-Junior

**Affiliations:** Universidade de São Paulo (USP), São Paulo, SP, Brazil

Teaching medical specialties is a challenge, as good doctors are not necessarily the best teachers. In this circumstance, good doctors, in general, have great technical knowledge about the specialty, but they are not always experts in methods of teaching.

In surgical teaching, the situation is even more difficult, as it requires technical knowledge, education methods, and manual skills. Thus, the professor of surgery would need, in addition to being a good surgeon, to know how to teach technique theory and how to transfer skills.

In technological surgeries, such as phacoemulsification cataract surgery, new technologies, which have brought more precision and safety to the procedure, have also made the Technique – Technology – Skill integration more complex.

In phacoemulsification, the surgeon who dominates the Technique and knows the Technology can reduce the influence of Skill on the surgical result. This is great news as Skill is a resource that needs practice and time to improve, depending, to some degree, on an individual condition (a “natural gift”). In this way, the diligent doctor, who studies and understands Technique and Technology, will be able to become a good surgeon in less time, not depending on having a “natural gift”. Just like doctors who had the opportunity to train their Skill, performing a very large number of surgeries and having a “natural gift” for manual maneuvers, they will become good surgeons, despite having studied little about the Technique and Technology.^1^^,^^2^

The difference the authors noticed in these two styles, of surgical teaching/learning, is that: 1) The surgeon who knows the Technique and Technology has better conditions to continuously improve his surgical performance than the one who basically depends on Skill; and 2) The surgeon who depends essentially on Skill will have more difficulty in transmitting his surgical knowledge to others and will limit himself to recommend the learner just to do what he does, as he may not have enough theoretical knowledge to explain “why” and “how” to do it. On the other hand, the surgeon who understands the Technique and knows the Technology will be better able to guide and correct the learning surgeon in each maneuver.^3^

However, having Skill, mastering the Technique, and knowing the Technology are still not enough for doctors to be good professors of surgery. The authors believe that this is a necessary, but not sufficient characteristic to be a good teacher. To be a professor of surgery, it is necessary to know how to teach. Didactic ability is a quality acquired through study and improved with practice, especially when considering active teaching methodologies.

Delegating the job of surgical teaching to good surgeons and older residents will provide only a “fireman” to resolve complications and not a professor of surgery. In this way, surgical learning will be longer, more “traumatic” and will predominantly depend on the student's proactivity.

In this circumstance, the authors believe that it is not enough for teaching hospitals to guarantee only good infrastructure for patients, exams, and surgeries. The authors also consider it important to invest in teacher training, avoiding the process of simply reproducing and transmitting knowledge in expositive classes.

In fact, for adequate surgical teaching, it is not enough to depend only on the instructive qualities of professors. It is important to guarantee the opportunity for the student to learn, providing time for theoretical teaching. The basis of teaching surgery must be in the classroom, through an explanation of the theory and discussion of videos of surgeries. Technique and Technology (Cognitive Domain) need to be taught in the amphitheater and not in the surgical center, no matter how good the professor is. Thus, there needs to be an effective theoretical knowledge transfer program, which should preferably be the responsibility of the professor of surgery, who uses modern instructive methodologies. The effective performance of the surgery, under supervision, is the final stage of learning, in which Manual Skill will be trained (Psychomotor Mastery) and the knowledge of Technique and Technology, put into practice.

Still, in the area of teaching models, the authors consider that transmitted information needs to be supported by the practice of Evidence-Based Medicine (EBM), and not be the result of individual perceptions alone.

An important talent for teachers is the ability to analyze critically and reflectively the information they will transmit. The doctor raised as an educator, needs to be aware that he is now an opinion-maker and that his words will have repercussions. It is therefore necessary to have a commitment to scientific “truth”. The “truth” based on personal experience is partial, as people “only know what they know”. Making a judgment based on just one aspect of the “truth” and applying it to the whole does not represent the real “truth”. The authors’ personal experiences are often misleading and incomplete because they are based on limited information. Although in science, the “truth” is always provisional, the scientific consensus remains the safest way to approach the truth.

There should be training for teachers on how to answer questions with a study of scientific articles in electronic databases, through the practice of EBM. Professors would need to have basic knowledge of clinical research, to train critical thinking. The practice of critical thinking builds the scientific method, which will help to discriminate between what is scientific fact and what is opinion.^4^

In the authors’ view, surgical teaching should not be totally free, informal, and according to the professor's convenience and the institution's opportunities. It is necessary to have a defined academic strategy so that learning is not essentially dependent on the proactivity of the student. This way, even limitations of the institution's resources could be minimized.

## Conflicts of interest

The author declares no conflicts of interest.
